# Coenzyme Q_10_: Clinical Applications in Cardiovascular Diseases

**DOI:** 10.3390/antiox9040341

**Published:** 2020-04-22

**Authors:** Alma Martelli, Lara Testai, Alessandro Colletti, Arrigo F. G. Cicero

**Affiliations:** 1 Department of Pharmacy, University of Pisa, 56120 Pisa, Italy; alma.martelli@unipi.it (A.M.); lara.testai@unipi.it (L.T.); 2 Interdepartmental Research Centre “Nutraceuticals and Food for Health (NUTRAFOOD)”, University of Pisa, 56120 Pisa, Italy; 3 Interdepartmental Research Centre of Ageing, Biology and Pathology, University of Pisa, 56120 Pisa, Italy; 4 Department of Science and Drug Technology, University of Turin, 10125 Turin, Italy; alessandro.colletti@unito.it; 5 Italian Nutraceutical Society (SINut), Via Guelfa 9, 40138 Bologna, Italy; 6 Medical and Surgical Sciences Department, University of Bologna, 40126 Bologna, Italy

**Keywords:** coenzyme Q_10_, ubiquinone, cardiovascular disease, risk factors, prevention, supplementation

## Abstract

Coenzyme Q_10_ (CoQ_10_) is a ubiquitous factor present in cell membranes and mitochondria, both in its reduced (ubiquinol) and oxidized (ubiquinone) forms. Its levels are high in organs with high metabolism such as the heart, kidneys, and liver because it acts as an energy transfer molecule but could be reduced by aging, genetic factors, drugs (e.g., statins), cardiovascular (CV) diseases, degenerative muscle disorders, and neurodegenerative diseases. As CoQ_10_ is endowed with significant antioxidant and anti-inflammatory features, useful to prevent free radical-induced damage and inflammatory signaling pathway activation, its depletion results in exacerbation of inflammatory processes. Therefore, exogenous CoQ_10_ supplementation might be useful as an adjuvant in the treatment of cardiovascular diseases such as heart failure, atrial fibrillation, and myocardial infarction and in associated risk factors such as hypertension, insulin resistance, dyslipidemias, and obesity. This review aims to summarize the current evidences on the use of CoQ_10_ supplementation as a therapeutic approach in cardiovascular diseases through the analysis of its clinical impact on patients’ health and quality of life. A substantial reduction of inflammatory and oxidative stress markers has been observed in several randomized clinical trials (RCTs) focused on several of the abovementioned diseases, even if more RCTs, involving a larger number of patients, will be necessary to strengthen these interesting findings.

## 1. Introduction

**Figure 1:**
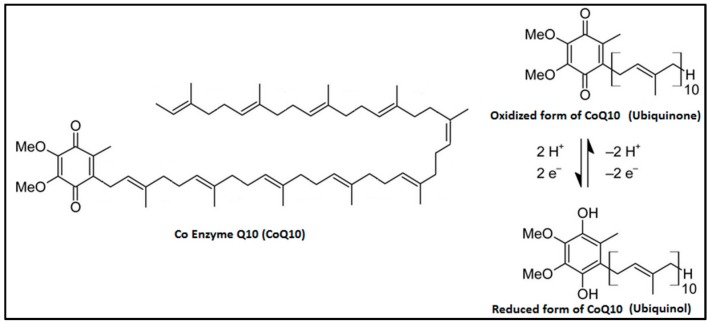
Chemical structure of CoQ_10_.

Coenzyme Q_10_ (CoQ_10_) is an organic molecule that was identified for the first time by Frederick Crane of Wisconsin (USA) in 1957 [1]. It is ubiquitously present in cell membranes and especially in the mitochondria in both reduced (ubiquinol) and oxidized (ubiquinone) forms (Figure 1). Chemically, it is constituted of a benzoquinone group and a poly-isoprenoid side chain that is species specific. In the human, it is composed of 10 units and called CoQ_10_ or ubiquinone [2]. This molecule can sustain continuous oxidation–reduction cycles and is an excellent electron carrier. CoQ_10_ concentration is particularly high in organs such as the kidneys, heart, and liver (Table 1) because they need it as an efficient energy transfer molecule supporting their high metabolic rate [3].

Physiologically, CoQ_10_ is anchored in the cell membrane through the isoprenoid tail, whereas the benzoquinone ring moves in the membrane based on its redox state. The most prominent role of CoQ_10_ is to facilitate the production of ATP through participation in the electron transport chain in the mitochondria. In fact, in the respiratory chain, CoQ_10_ transfers electrons from complex I (nicotinamide-adenine dinucleotide (NADH)-coenzyme Q reductase) or complex II (succinate-coenzyme Q reductase) to complex III (cytochrome c reductase), and it is also a structural component of both CI and CIII, reducing the production of reactive oxygen species (ROS) [6,7].

Moreover, CoQ_10_ is able to accept electrons from fatty acyl-coenzyme A (acyl-CoA) dehydrogenases and it is an obligatory factor in proton transport by uncoupling proteins (UCPs), thus regulating the opening of mitochondrial permeability transition pores [8]. Other functions of CoQ_10_ in the cell membrane include stabilization of calcium-dependent channels, metabolic regulation, cell signaling, and cell growth through local regulation of cytosolic redox intermediates such as dihydronicotinamide-adenine dinucleotide phosphate (NADPH) [6].

CoQ_10_, in its reduced form, has been shown to inhibit the peroxidation of cell membrane lipids and to reduce the oxidation of circulating lipids. Interestingly, in vitro, it inhibits the oxidation of low-density lipoprotein more than other antioxidant molecules, such as α-tocopherol or β-carotene [9,10].

CoQ_10_ is mostly synthetized in the cell, although the pathway involved is not yet completely known. A biosynthetic complex for producing CoQ_10_, containing proteins, lipids, and polar small molecules (but with specific composition unknown), was recently revealed in yeast and mammals. In particular, multiple mitochondrial uncharacterized proteins (MXPs) have been linked to CoQ_10_ biosynthesis and recent progress was made also toward understanding the biochemistry of a dehydrogenase, a deaminase, a lipid-binding protein, and a protein kinase-like enzyme in the CoQ_10_ pathway [11]. In mammalians, 4-hydroxybenzoate is the precursor of the quinone ring, derived from tyrosine, while the isoprenoid tail is derived from the mevalonate pathway, using the common way with cholesterol biosynthesis. The final step, rate limiting, occurs in the mitochondrial matrix [12,13].

On the other hand, CoQ_10_ can be derived from the diet; in particular, fatty fish (salmon, sardin, and tuna), soya, spinach, and nuts contain high levels of this cofactor. However, the intake from the diet is significant only in deficiency conditions [14]. Some factors may reduce plasma concentrations of CoQ_10_, such as aging, genetic factors, drugs (e.g., statins), certain diseases (e.g., cardiovascular disease and degenerative muscle disorders), and increased demand [15].

Therefore, it is not surprising that its depletion is associated with a greater propensity to develop immune inflammatory responses through the activation of inflammatory processes such as the nuclear factor-kappa-light-chain-enhancer of activated B cell’s (NF-κB) gene expression [16]. Worthy to note, CoQ_10_ is endowed with potent antioxidant action able to prevent free radical damage by the regulation of transcriptional pathways in addition to deactivation of inflammatory pathways [17]. Therefore, supplementation with CoQ_10_ could be efficient in the prevention and/or treatment of a number of pathogenic disorders in relation to the significant reduction of inflammatory markers [18].

Due to its important place in organisms’ functioning, there are many diseases and degenerative states associated with CoQ_10_′s deficiency, such as cardiovascular disease, muscular dystrophy, Alzheimer’s disease, Parkinson’s disease, and others [7]. However, if on the one hand clinical evidences in the cardiovascular field have demonstrated the potential role of CoQ_10_, data concerning the supplementation of this nutraceutical in neurodegenerative diseases and other conditions such as cancer or muscular dystrophy are often old and still conflicting and need additional randomized controlled trials (RCTs) [19,20,21].

This review aims to sum up the current possibilities to use CoQ_10_ as an adjuvant in cardiovascular disease-affected patients, in cardiovascular disease risk factors, and in statin-intolerant ones, with an analysis of its impact on patients’ health and quality of life.

## 2. Methods

A systematic search strategy was conducted for this review in order to identify trials in both the Cochrane Register of Controlled Trials (The Cochrane Collaboration, Oxford, UK) and MEDLINE (National Library of Medicine, Bethesda, Maryland, MD, USA; January 1970 to March 2020). The terms “coenzyme Q_10_”, “dietary supplement”, “ubiquinol”, “ubiquinone”, “clinical trial”, and “human” were incorporated in an electronic search strategy. Overall, we screened 5278 abstracts. The selected references were then further screened for application on cardiovascular diseases or cardiovascular disease risk factors. After a general introduction with an overview on the pharmacodynamic profile of CoQ_10_, for each potential therapeutic indication, a short description of the mechanism of action has been reported, followed by the clinically observed effects and the most relevant tolerability notes. The authors of the writing and reviewing panels completed *Declaration of Interest* forms where real or potential sources of conflicts of interest might be perceived.

## 3. Results

**Figure 2:**
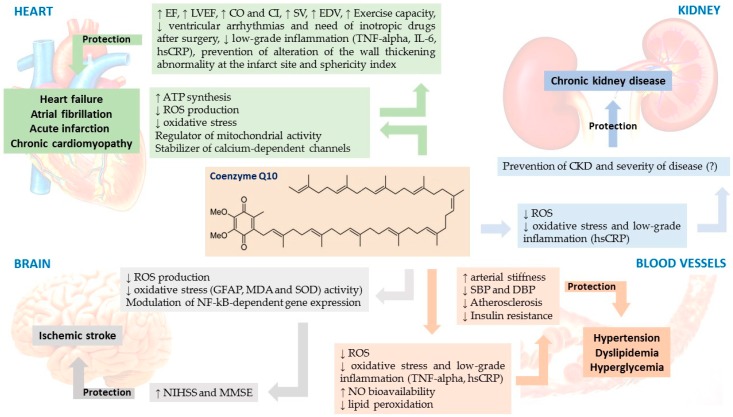
Involvement of CoQ_10_ deficiency and cardiovascular disease risk factors. ATP: adenosine triphosphate; CI: cardiac input; CO: cardiac output; CKD: chronic kidney disease; DBP: diastolic blood pressure; EDV: end-diastolic volume; EF: ejection fraction; GFAP: glial fibrillary acidic protein; hs-CRP: high sensible- C reactive protein; IL-6: interleukin-6; LVEF: left ventricular ejection fraction; MDA: malondialdehyde; MMSE: mini mental state examination; NIHSS: national institute of health stroke scale; NO: nitric oxide; NF-kB: nuclear factor kappa B; ROS: reactive oxygen species; SBP: systolic blood pressure; SOD: superoxide dismutase; SV: stroke volume; TNF-alpha: tumor necrosis factor-alpha.

This review will focus our attention on the main potential evidence-based use of CoQ_10_ supplements in the management of some main cardiovascular disease risk factors and of cardiovascular disease-affected patients and in statin-intolerant ones (Figure 2).

### 3.1. CoQ_10_ and Cardiovascular Risk Factors

As stated above, CoQ_10_ supplementation could find a role in the management of some highly prevalent cardiovascular and cerebrovascular disease risk factors, such as high blood pressure, insulin resistance, dyslipidemia, migraine, and chronic kidney disease.

#### 3.1.1. High Blood Pressure

Hypertension is one of the major causes of morbidity and mortality worldwide, involving one in four men and one in five women, totalling 1.13 billion adults, who had raised blood pressure in 2015 [22]. A recent comparative assessment of the risk of health loss related to systolic blood pressure (SBP), based on 844 studies in 154 countries (published between 1980 and 2015) and 8.69 million participants, has estimated approximately 874 million of people in the world with SBP above 140 mmHg [23]. In 2025, it is estimated that there will be approximately 1.56 billion hypertensive adults [24].

CoQ_10_ seems to exert a direct effect on the endothelium, provoking vasodilation and lowering blood pressure [25,26]. This effect is linked to its ability to improve nitric oxides bioavailability and to induce vasodilatation especially in patients with hypertension. In addition, CoQ_10_ adjusts the angiotensin effect in sodium retention and decreases the level of aldosterone [27,28]. Despite exciting blood pressure results observed in preliminary trials (systolic and diastolic blood pressure reduced respectively by 6 and 5 mmHg vs. placebo) [29] and the positive results confirmed by old meta-analyses of RCTs [30,31], a recent meta-analysis of 17 randomized controlled trials including 684 subjects showed that CoQ_10_ supplementation significantly decreased systolic blood pressure (Standardized Mean Difference (SMD) −0.30; 95%CI −0.52, −0.08), but not diastolic blood pressure (SMD −0.08; 95%CI −0.46, 0.29) [32]. However, in patients with type 2 diabetes mellitus and ischemic left ventricular systolic dysfunction, when the blood pressure is on target, the supplementation of CoQ_10_ did not modify the blood pressure [33,34,35]. In conclusion, despite some promising evidence, the antihypertensive effect of CoQ_10_ is still unclear in patients with primary hypertension [36,37].

#### 3.1.2. Insulin-Resistance and Type 2 Diabetes

Mitochondria seem to play a key role in the development of insulin resistance. They are well known to convert nutrients from diet such as fats and sugars into ATP; however, ATP production can generate harmful intermediates such as ROS and the increase in the amount of oxidant agents produced in mitochondria has been linked to the increase of insulin resistance [38,39]. Several studies in vitro and in vivo as well [40] found that the concentrations of CoQ_10_ were lower in mitochondria from insulin-resistant fat and muscle tissue, probably for a change in expression of mevalonate/CoQ_10_ pathway proteins and thus altered CoQ_10_ metabolism, suggesting a direct correlation between the low levels of CoQ_10_ and the high levels of oxidants in the mitochondria. In addition, the administration of CoQ_10_ in deficient and insulin resistant mice has been shown to improve the insulin sensitivity by reducing ROS levels [40].

In patients with metabolic syndrome (MetS), a condition typically caused by insulin-resistance and strongly associated with the risk to developing cardiovascular disease, the intake of 100 mg/day of CoQ_10_ for 8 weeks significantly improved Homeostatic Model Assessment of Insulin Resistance (HOMA-IR), Homeostatic Model Assessment of β-cell Function (HOMA-B), serum insulin levels, and plasma total antioxidant capacity [41]. The effect of CoQ_10_ on insulin-resistance seems to not be related to its effect on body fat. In fact, a recent meta-analysis of RCTs showed that CoQ_10_ had no significant impact on body weight (*p* = 0.64) and body mass index (BMI) (*p* = 0.86), independent from the CoQ_10_ tested dosage and trial duration [42].

Another highly prevalent cardiovascular risk factor related to insulin-resistance is nonalcoholic fatty liver disease (NAFLD) [43]. Despite the numerous mechanisms investigated, the exact biological one related to increased hepatic inflammation and fat accumulation in NAFLD remains largely unknown [44,45]. However, recent studies have focused attention on the role of mitochondrial protein mitofusin 2 (Mfn2) that protects against liver disease. In fact, reduced Mfn2 expression was detected in liver biopsies from patients with nonalcoholic steatohepatitis [46]. The loss of Mfn2 seems to impair mitochondrial respiration and to reduce ATP production, and this defective oxidative phosphorylation process seems to unexpectedly originate from a depletion of the mitochondrial CoQ_10_ pool [47].

To date, the treatment of NAFLD is essentially based on lifestyle optimization because there are currently no specific drugs approved on the market for this condition. At the same time, few nutraceuticals have been adequately studied for their effects on NAFLD [48]. Among these, CoQ_10_ is a well-known anti-adipogenic molecule and thus could have a positive impact on NAFLD, even if its exact mechanism is still unclear. It is possible that CoQ_10_ downregulates the expression of fatty acid synthase (FAS), sterol regulatory element-binding protein-1c (SREBP-1c), and acetyl-CoA carboxylase (ACC), which are related to lipid synthesis, and increases in the expression of carnitine palmitoyltransferase-1 (CPT-1) and peroxisome proliferator-activated receptors α (PPARα) associated with fatty acid oxidation [49]. In addition, CoQ_10_ could change the response to inflammation through nuclear factor kappa B (NF-kB)-dependent gene expression [50]; thus, its deficiency might have a role in increasing levels of inflammatory molecules like NF-kB [51].

CoQ_10_ could serve as an adenosine monophosphate-activated protein kinase (AMPK) activator and could regulate the hepatic lipid metabolism to inhibit the abnormal accumulation of hepatic lipids as well as to prevent NAFLD progression [49]. Finally, CoQ_10_ was also found to bind and activate both PPARs alpha and gamma, suggesting a key role in relaying the states of mitochondria and peroxisomes [52]. At the same time, the experiments performed with peroxisomal inducers indicate that nuclear receptors are involved in the regulation of CoQ_10_ biosynthesis [13].

In an RCT, 41 subjects with NAFLD were randomly divided into 2 groups to receive CoQ_10_ (100 mg/day) or placebo for 12 weeks. At the end of the study, the active group benefited from a significant decrease in aspartate aminotransferase (AST), gamma-glutamyl transpeptidase (GGT), tumor necrosis factor α, high-sensitivity C-reactive protein (hs-CRP), and NAFLD grade compared to placebo (*p* < 0.05 for all). In addition, patients who received the CoQ_10_ supplement had higher serum levels of adiponectin (*p* = 0.016) even if serum leptin levels reduced marginally (*p* = 0.053) [53]. However, CoQ_10_ administration (300 mg/day for 12 weeks) in patients with coronary artery disease did not find any significant effect on serum adiponectin levels [54], confirming previous data obtained by Gokbel et al. with the supplementation of CoQ_10_ 100 mg/day in healthy volunteers [55]. In another RCT, the same dose of CoQ_10_ in 44 NAFLD patients for 4 weeks was associated with significantly decreased waist circumference (WC), serum AST, and total antioxidant capacity (TAC) concentration (*p* < 0.05 for all) [56].

CoQ_10_ could also improve the atherogenic dyslipidemia typically associated with NAFLD (reducing triglycerides (TG) and increasing high-density lipoprotein cholesterol (HDL-C) as well as reduce oxidized low-density lipoprotein (LDL) levels and arterial pressure with a very high safety profile and without any risk of drug interactions [15]. In conclusion, the studies conducted to date emphasize a potential for CoQ_10_ therapy in improving several anthropometric and biochemical variables in NAFLD.

A further disease typically characterized by insulin resistance is polycystic ovary syndrome (PCOS). In these women, as showed by the study of Samimi et al., the supplementation with CoQ_10_ (100 mg/day) for 12 weeks could have beneficial effects on glucose metabolism and on serum total- and LDL-cholesterol levels [57]. Afterwards, the same research group carried out another RCT on 40 women with a diagnosis of PCOS, observing that a supplementation for 12 weeks with CoQ_10_ (100 mg/day), beside the positive effects on lipid and glucose levels, was responsible for a downregulation of gene expression of oxidized low-density lipoprotein (LDL) receptor 1 (*p* < 0.001) and an upregulated gene expression of PPAR-γ (*p* = 0.01) in peripheral blood mononuclear cells. In addition, compared to the placebo group, CoQ_10_ supplementation downregulated gene expression of interleukin-1 (IL-1) (*p* = 0.03), IL-8 (*p* = 0.001), and tumor necrosis factor-alpha (TNF-α) (*p* < 0.001) in peripheral blood mononuclear cells of subjects with PCOS [58]. Similar results were obtained by Izadi et al. in a RCT of 85 PCO women treated with CoQ_10_ and/or vitamin E or placebo. In particular, CoQ_10_ alone improved the sex homone profile, specially either reduced testosterone and luteinizing hormone (LH) levels, and improved insulin resistence. Moreover, it is noteworthy that CoQ_10_ in coadministration with alfa-tocopherol presented a more pronunced effect and stimulated the release of sex hormone-binding globulin (SHBG), justifing the enhancement of insulin tolerance, since an insulin resistance condition is associated with a reduced synthesis of SHBG at the hepatic level. Then, CoQ_10_ might promote steroid hormone biosynthesis and normal reproductive function (among which are oocyte maturation, fertilization, and embryonic development) through the improvement of mitochondrial functionality [59]. However, new, larger RCTs are needed to confirm the results obatined by Izadi et al.

The extreme consequence of insulin-resistance is Type 2 diabetes (T2DM). A deficiency of CoQ_10_ plasma levels in patients with T2DM can be observed compared to healthy people [60,61]. In particular, the ubiquinone–ubiquinol ratio, a validated marker of oxidative stress [62], is much higher in a patient with T2DM after breakfast and throughout the day, which suggests heightened oxidative stress in the background of postprandial hyperglycemia [63]. In a recent pooled analysis of 14 trials including 693 overweight diabetic patients, CoQ_10_ interventions significantly reduced fasting plasma glucose (FPG) (−0.59 mmol/L; 95%CI −1.05 to −0.12; *p* = 0.01), HbA1c (−0.28%; 95%CI −0.53 to −0.03; *p* = 0.03), and TG levels (0.17 mmol/L; 95%CI −0.32 to −0.03; *p* = 0.02). Even in the subgroup analysis, the low-dose consumption of CoQ_10_ (<200 mg/d) effectively reduced the values of FBG, HbA1c, fasting blood insulin, homeostatic model assessment for insulin resistance (HOMA-IR), and TG with high tolerability profile [64]. In a rat model, the administration of metformin combined with CoQ_10_ showed a better renoprotective effect than CoQ_10_ or metformin alone [65]. This is also confirmed for other oral antidiabetic drugs like sitagliptin [66]. This brings up an important point that CoQ_10_ may potentiate the protective effects of some conventional treatments, but it is yet to be demonstrated in humans.

#### 3.1.3. Dyslipidemias

Several mechanisms have been proposed by which CoQ_10_ supplements could improve metabolic profiles which probably might be through the induction of gene expression of PPAR-γ [67], a nuclear receptor protein that regulates gene expression involved in insulin and lipid metabolism, differentiation, proliferation, survival, and inflammation [68]. In human endothelial cells, the exposure to CoQ_10_ is associated with downregulation of the lectin-like oxidized LDL receptors, stimulation of the AMPK, and reduction of the ROS-induced endothelial damage [69]. In fact, the main effect of CoQ_10_ on plasma lipids seems to be the increased LDL resistance to oxidative stress [70], as also demonstrated in healthy adults after acute strenous physical exercise [71]. 

In an RCT, 101 dyslipidemic subjects without taking any lipid-lowering drugs were administrated 120 mg CoQ_10_ or placebo daily for 24 weeks. At the end of the study, CoQ_10_ supplementation mildly reduced TG (*p* = 0.020) and LDL-C (*p* = 0.016), increased apolipoprotein (Apo)A-I (*p* < 0.001) and serum total antioxidant capacity (TAC; *p* = 0.003), while decreased homeostasis model assessment of insulin resistance index (*p* = 0.009) compared to placebo [24]. In the meta-analysis conducted by Sharifi et al. [72], CoQ_10_ administration to patients with metabolic diseases mildly but significantly reduced TG concentrations (SMD −0.28 mmol/L; 95% CI, −0.56 to −0.005, *p* = 0.001). A recent meta-analysis including six clinical trials suggests that CoQ_10_ could mildly reduce the lipoprotein (a) plasma level [73]. Overall, the effect of CoQ_10_ supplementation on plasma lipid levels is, however, quantitatively small and its clinical relevance has yet to be demonstrated.

#### 3.1.4. Systemic Inflammation

Inflammation is considered a main process involved in atherosclerosis development [74]. A recent meta-analysis of nine RCTs and 509 patients showed that the CoQ_10_ supplementation in chronic inflammatory diseases (60–500 mg/day for 8–12 weeks) is responsible for the significant reduction in the plasma levels of tumor necrosis factor alpha (TNF-α) (SMD: −0.44, 95% CI: (−0.81 to −0.07) mg/dl; *I*^2^ = 66.1%, *p* < 0.01) and in IL-6 levels (SMD: −0.37, 95% CI: (−0.65 to −0.09), *p* = 0.01) [75]. Similar results were obtained by the metanalysis of Fan et al. that demonstrated a reduction of the C-reactive protein levels in addition to the abovementioned parameters in patients afflicted by inflammatory diseases [76]; in elderly people with low CoQ_10_ levels; and in patients with metabolic diseases characterized by chronic, low grade inflammation [17]. However, the results are conflicting while not so evident in patients affected by metabolic syndrome [41] and dyslipidemia [29].

### 3.2. CoQ_10_ and Cardiovascular Disease

CoQ_10_ supplementation has been tested in a number of overt cardiovascular diseases, with the aim to evaluate its impact on self-perceived quality of life, instrumental parameters, and sometimes clinical outcomes as well.

#### 3.2.1. CoQ_10_ and Heart Failure (HF)

HF is defined by the American Heart Association (AHA)/American College of Cardiology (ACC) guidelines as “a complex clinical syndrome that can result from any structural or functional cardiac disorder that impairs the ability of the ventricle to fill or eject blood” [77,78]. It affects 23 million people worldwide [79], and the HF prevalence in the USA is 5 million people [80]. At the same time, this disease is also the main component for disability and hospitalization in the elderly and it is the cause of one in nine deaths in the USA [1]. In Europe, the prevalence and incidence of HF and the related costs are quite similar [81,82]. Despite that, in the last decades, the prevention and treatment of HF have improved significantly, quality of life is often impaired, and mortality rates are greater than 10% per year, reaching 20%–50% in more serious patients [83]. In the last years, a number of clinical studies have investigated the possibility that CoQ_10_ can contribute to the prevention of incident HF and to the improvement of related symptoms and instrumental parameters. Being an essential cofactor of the mitochondrial respiratory chain used for production of adenosine triphosphate (ATP), it is not surprising that the highest concentration compared to other tissues is focused on myocardium mitochondria [84].

A relative tissue CoQ_10_ deficiency could then play an etiopathogenic role in the development and progression of HF: some evidence suggests that the depletion of CoQ_10_ is proportional to the reduction of CoQ_10_ myocardial tissue concentrations and to the severity of the disease developed [85,86,87]. In fact, the lowest levels of myocardial CoQ_10_ have been observed in patients of New York Heart Association (NYHA) class IV compared to patients of NYHA class I [88,89]. Of course, one of the most important studies in the field of nutraceuticals, the Q-SYMBIO multicentre, randomized placebo-controlled trial, was used to assess the impact of the daily intake of CoQ_10_ on total mortality and not just on the surrogate endpoints. Patients with moderate or severe HF currently treated with the pharmacological gold standard treatments (420 patients) were randomized to a daily intake of 300 mg of CoQ_10_ (*n* = 202) or placebo (*n* = 218). After two years, a significant reduction in Major Adverse Cardiac Events (MACE) rate (15% in the CoQ_10_ group vs. 26% in the placebo group, HR: 0.50; 95%CI: 0.32 to 0.80; *p* = 0.003), CV mortality (9% vs. 16%, *p* = 0.026), all-cause mortality (10% vs. 18%, *p* = 0.018), and incidence of hospital stays for HF (*p* = 0.033) were registered in CoQ_10_-treated patients vs. the placebo treated ones [90]. This result was confirmed in a subsequent meta-analysis of 14 RCTs including 2149 patients. It has shown that administration of CoQ_10_ reduces mortality (RR= 0.69; 95%CI: 0.50–0.95; *p* = 0.02; *I*^2^= 0%) and improves exercise capacity (SMD = 0.62; 95%CI: 0.02–0.30; *p* = 0.04; *I*^2^= 54%) compared to the placebo. However, no significant difference was observed in the endpoints of left ventricular ejection fraction (LVEF) between “active group” and placebo (SMD = 0.62; 95% CI: 0.02–1.12; *p* = 0.04; *I*^2^ = 75%) [91]. The effect on LVEF could be more relevant in patients with preserved ejection fraction (EF) [92] (net change: 4.8% vs. subjects with EF < 30%) and patients untreated with statins and/or angiotensin converting enzyme inhibitors (ACEi) (+6.7%) compared to the subgroup of patients treated with these drugs (+1.2%) [93]. One of the possible explanations of the heterogeneity in results on EF may be the diversity of CoQ_10_ supplemented through different pharmaceutical forms and dosages. In fact, plasma concentrations of this molecule are extremely variable in relation to pharmaceutical form and administered dosages but were reported in few RCTs [94,95,96]. In addition, the diversity of HF grade of patients enrolled (NYHA I-II-III-IV), duration of treatments, and cotreatment with conventional therapies might be other factors that could explain the heterogeneity of results about EF [97].

#### 3.2.2. CoQ_10_ and Myocardial Infarction

HF could be related to different causes: one of the most frequent is ischemic damage. As highlighted before, treatment with CoQ_10_ in HF could prevent myocardial cell damage and could restore tissue CoQ_10_ deficiency, especially in myocardial tissue, with the final result being significant improvement in HF [98,99,100,101]. The degree of deficiency of this molecule has also been found to correlate directly with the degree of impairment in left ventricular function [102]. For these reasons, another possible indication of CoQ_10_ supplementation is acute myocardial infarction (AMI). In fact, CoQ_10_ is an ATP-sparing agent and regenerable antioxidant capable of protecting cell structures from oxidative damage during ischemia and reperfusion injury [103,104].

AMI is typically characterized by complications such as left ventricular dysfunction related to necrosis and loss of functioning myocardium and consequently by pathological remodelling, which seem to be related to reperfusion-induced free radical damage, lipid peroxidation, and decreased energy production and thus the lack of CoQ_10_ [105,106,107,108]. Cardiac remodelling may be defined as “a group of molecular, cellular, and interstitial alterations that manifest clinically as changes in size, mass, geometry, and function of the heart after injury” [105]. These structural changes in ventricular remodelling in conjunction to tissue CoQ_10_ deficiency may result in poor prognosis for its negative association with HF, which is the major cause of morbidity and mortality in patients with AMI [109]. *O*xidative stress may be important in the pathogenesis of remodelling which may begin via subcellular remodelling leading to HF [110]. Therefore, any agent which can prevent remodelling in patients with AMI would be an important therapeutic aid for prevention of complications altering AMI [111,112]. In a recent RCT of 55 patients with LVEF < 50% after AMI, the effects of CoQ_10_ (120 mg/day) or placebo were studied for 24 weeks. The results revealed that wall thickness opposite the site of infarction decreased from 12.2 ± 2.0 mm to 10.0 ± 1.8 mm with CoQ_10_ compared with 12.8 ± 2.2 mm to 13.3 ± 2.3 mm with the placebo (*p* < 0.01). Left ventricular mass changed from 236 ± 72 g to 213 ± 61 g with CoQ_10_ compared with 230 ± 77 g to 255 ± 86 g with placebo (*p* < 0.01). In addition, treatment with CoQ_10_ also prevented alteration of the sphericity index (from 1.61 ± 0.28 to 1.63 ± 0.30 with CoQ_10_ compared with 1.61 ± 0.32 to 1.41 ± 0.31 with placebo (*p* < 0.05)) and alteration of the wall thickening abnormality at the infarct site (from 9.4 ± 3.0 cm^2^ to 9.1 ± 2.8 cm^2^ compared with 10.1 ± 3.1 to 13.7 ± 4.2 cm^2^ with placebo (*p* < 0.05)). Finally, end diastolic and systolic volumes and serum ACE also showed significant reduction with CoQ_10_ compared to the control group [107]. The findings suggest that CoQ_10_ administered early after AMI may be protective against left ventricular remodelling in patients with persistent left ventricular dysfunction. However, long-term RCTs are needed to confirm preliminary data.

#### 3.2.3. CoQ_10_ and Atrial Fibrillation

Atrial fibrillation (AF) is considered a frequent atrial arrhythmia in patients diagnosed with HF or ischemic heart disease, and its prevalence has been growing worldwide in the last years. It is associated with an increase in morbidity and mortality [113,114,115]. As underlined for HF, CoQ_10_ plays an important role in the production of ATP and its bioenergetic function associated to with antioxidant and scavenge ROS function which is essential for proper heart functioning [116,117]. A meta-analysis of eight RCTs found that patients treated with CoQ_10_ were significantly less likely to develop ventricular arrhythmias (OR (95% CI) 0.05 (0.01–0.31)) and to require inotropic drugs after surgery (OR 95% CI 0.47 (0.27–0.81)). Twelve patients (22.2%) in the control group and three patients (6.3%) in the CoQ_10_ group had episodes of AF after 12 months of treatment (*p* = 0.02). [118] Similar results were obtained by other authors, concluding that CoQ_10_ as adjuvant treatment in patients with HF may attenuate the incidence of AF. The exact mechanisms of the effect are still unclear, even if one of the possible explanations could be attributed to the reduction of serum levels of malondialdehyde (MDA) [119].

#### 3.2.4. CoQ_10_ and Nonischemic Cardiomyopathies

Cardiomyopathies are a number of debilitating conditions responsible for poor quality of life and high risk of mortality. Both in vitro and animal studies suggest a link between cardiomyopathies and oxidative stress [120]. CoQ_10_ deficiency appears to be frequent in people with dilated cardiomyopathy, and its supplementation may be able to restore plasmatic and myocardial levels [121]. However, new studies are needed to confirm this evidence.

In children with dilated cardiomyopathy, CoQ_10_ may improve the cardiothoracic ratio and shorten ventricular depolarization and NYHA class [122]. In a prospective RCT (duration 6 months) in children with dilated cardiomyopathy, the administration of CoQ_10_ resulted in a lower mean score for the index of cardiac failure (*p* < 0.024 compared to placebo) and in improvement of diastolic function (*p* < 0.011 compared to placebo) [123]. In subjects with hypertrophic cardiomyopathy treated with an average of 200 mg/day of CoQ_10_, a significant improvement in symptoms of fatigue and dyspnoea with no side effects was noted. In addition, the mean interventricular septal thickness (from 1.51 ± 0.17 cm to 1.14 ± 0.13 cm, a 24% reduction, *p* < 0.002) and mean posterior wall thickness improved significantly (from 1.37 ± 0.13 cm to 1.01 ± 0.15 cm, a 26% reduction, *p* < 0.005) [124]. There is also a significant improvement in quality of life (on a 6-min walk test) and NYHA class (≥1) [125].

In the last years, many studies have focused on the role of CoQ_10_ in iatrogenic cardiomiopathies induced by some drugs like anthracycline antibiotics used in the chemotherapy of hematological cancers as leukemias and lymphomas and in solid malignancies such as carcinomas and sarcomas [126]. Doxorubicin is used for the treatment of early-stage breast cancer, and it is known to improve overall survival. However, side effects such as cardiomyopathy and HF can occur in some patients, probably also for a raised ROS generation. Today, there is data indicating that CoQ_10_ did not have any influence on doxorubicin cell toxicity, thus making further studies urgent [127]. Nevertheless, the administration of CoQ_10_ and L-carnitine in combination showed protection against oxidative stress by reducing levels of malondialdehyde and nitric oxide if started within 5 days before doxorubicin use. In addition, it also improved heart functions and decreased IL-1 and TNF-α Troponin-l and Troponin-T levels [128].

#### 3.2.5. CoQ_10_ and Ischemic Stroke

In the pathophysiology of ischemic stroke, some factors such as inflammation, excitotoxicity, and oxidative stress were demonstrated to play a pivotal role [129,130]. A recent study demonstrated the decrement of CoQ_10_ in the acute phase of ischemic stroke and also the significant negative correlation between serum CoQ_10_ levels and the scores of the NIHSS and MRS (respectively National Institutes of Health Stroke Scale and Modified Ranking Scale) [131]. Ischemia/Reperfusion (I/R) injury may induce oxidative stress and low levels of protective antioxidants such as CoQ_10_ in the brain. In particular, it seems that a decrease of CoQ_10_ induced by I/R overcomes the aging process [132]. In vivo studies (with symptomatic vasospasm model) have reported that pretreatment with CoQ_10_ reduces the incidence of ischemic lesions and can alleviate the pathological outcomes following a stroke incidence [133].

In the last years, the relation between CoQ_10_ and inflammation and oxidative stress has been reported in cell and animal models. Glial fibrillary acidic protein (GFAP), MDA, and superoxide dismutase (SOD) activity are important biomarkers in oxidative stress and neuroinflammatory processes after stroke, and they can predict functional outcomes [134,135,136]. In a short RCT, 60 patients with acute ischemic stroke were randomly assigned to a placebo or CoQ_10_-supplemented group (300 mg/day) for 4 weeks. At the end of treatment, CoQ_10_ supplementation improved NIHSS and MMSE scores significantly (*p* = 0.05, *p* = 0.03 respectively) even if there were no significant differences in MRS score, SOD, MDA, and GFAP levels between the two groups. These results could be partially explained by the low dose and short duration of supplementation [137].

### 3.3. Special Conditions

CoQ_10_ supplementation has been tested also in a number of “special conditions”, with the aim to evaluate its impact on self-perceived quality of life, instrumental parameters, and sometimes clinical outcomes as well.

#### 3.3.1. Chronic Kidney Disease

Chronic kidney disease (CKD) is associated with an increased prevalence of all-cause mortality, cardiovascular events and hospitalization, and diabetic nephropathy, all regardless of existing risk factors and a history of cardiovascular disease (CVD) [138,139]. Increased biomarkers of oxidative stress in these patients have been identified as a major contributor to the pathogenesis of CKD and related CVD [140,141]. Circulating concentrations of CoQ_10_ have been decreased in patients with CKD, suggesting that CoQ_10_ supplementation may be a potentially useful antioxidant supplement for these patients [142]. Nevertheless, the relation between CoQ_10_ and oxidative stress in patients with CKD is still controversial.

A meta-analysis of seven RCTs demonstrated that CoQ_10_ supplementation to patients with CKD significantly reduced total cholesterol (TC) (SMD = −0.58; CI, −0.94, −0.21; *p* = 0.002), LDL-C (SMD = −0.47; 95% CI, −0.78, −0.17; *p* = 0.003), malondialdehyde (MDA) (SMD = −3.0; 95% CI, −5.10, −0.90; *p* = 0.005), and creatinine levels (SMD = −1.65; 95% CI, −2.75, −0.54; *p* = 0.003) yet did not affect fasting glucose, insulin, HOMA-IR, and C reactive protein (CRP) concentrations [143]. Moreover, in a study not included in the previously cited meta-analysis, CoQ_10_ supplementation at a dosage of 100 mg/day for 12 weeks had positive effects on insulin metabolism and MDA levels among diabetic nephropathy patients yet fasting glucose remained unchanged [144]. Finally, a recent meta-analysis of 4 RCTs and 4 experimental studies of diabetic people revealed that CoQ_10_ combined with antidiabetic drugs show statistical differences in FPG (SMD = −2.04, 95% CI = −3.90 to −0.18, *p* < 0.05), TC (Std. MD = −1.73, 95% CI = −3.41 to −0.05, *p* < 0.05), HDL-C (Std. MD = 0.09, 95% CI = 0.01–0.18, *p* < 0.05), TG (Std. MD = −0.39, 95% CI = −0.71 to −0.07, *p* < 0.05), and MDA (Std. MD = −1.29, 95% CI = −2.32 to −0.26, *p* < 0.05) amelioration after diabetic kidney disease therapy compared to the control group [145].

CoQ_10_ supplementation for diabetic hemodialysis patients for 12 weeks did not influence lipid profiles [70,146,147]. In hemodialysis patients, 100 mg/day of CoQ_10_ for 3 months could significantly reduce CRP levels (95%CI = −20.1 to −10.5, *p* < 0.001) [148], while daily supplementation with 1200 mg of CoQ_10_ significantly improved biomarkers of oxidative stress [149].

Finally, the supplementation of CoQ_10_ could have a positive impact in people with nephrotic syndrome caused also by a subgroup of mitochondrial diseases classified as primary CoQ_10_ deficiency (pathogenic variants in at least one of 10 genes termed *COQ1* through *COQ_10_*). In contrast to other mitochondrial disorders, some patients with primary CoQ_10_ deficiency show significant improvements after CoQ_10_ supplementation, making early diagnosis and treatment essential in the management of these people [150].

#### 3.3.2. Migraine

Migraine is an emerging risk factor for both coronary and cerebrovascular diseases [151], for which the pathophysiology has not yet been fully understood. Among other factors, a deficiency of CoQ_10_ is associated with the pathogenesis of migraine, specially in pediatric and adolescent populations [152].

A systematic review and dose-response meta-analysis has been performed evaluating four RCTs including 221 subjects. CoQ_10_ significantly reduced the frequency of migraine attack (*p* < 0.001); however, no significant effect on severity and duration has been observed (*p* = 0.105 and *p* = 0.086, respectively) [153]. A more recent, larger meta-analysis of three RCTs and two observational studies, including 346 patients (120 pediatric and 226 adult subjects), has been carried out. In particular, with a daily dosage of CoQ_10_ of 100 or 400 mg, the forest plot analysis confirmed a significant reduction of the duration of migraine attack/month (*p* < 0.00001) and of the migraine day/month (*p* = 0.009) if compared with placebo. Nevertheless, frequency and severity of attacks (*p* = 0.08) were not changed [154].

Based on this data, the American Academy of Neurology guidelines suggest a possible role of CoQ_10_ in migraine prevention, with a high safety profile in pediatric and adult populations [155].

In one double-blind placebo controlled clinical trial on 45 patients (22 treated with placebo and 23 treated with CoQ_10_ at a dose of 400 mg/day for 3 months), a significant prophilactic effect of the supplementation on migraine attacks was reported, resulting in less severe, shorter, and less frequent attacks. Interestingly, an increase in serum levels of CoQ_10_ and a reduction of TNFα and calcitonin gene-related peptide (GCPR) levels have also been observed, suggesting a role of CoQ_10_ as mitigation of inflammatory processes [156]. According to other studies [43,157], however, no significant differences in serum IL6 and IL10 have been observed compared with the control groups [83].

Interesting results emerge by co-supplementation of CoQ_10_ (100 mg/day) with other nutraceuticals, such as curcumin, magnesium, and *Tanacetum parthenium* L. and riboflavin. In particular, Gaul and collaborators observed on 173 adults affected by migraine that a fixed combination of magnesium (600 mg/day), riboflavin (400 mg/day), and CoQ_10_ (150 mg/day) after 3 months of treatment reduced migraine pain without any serious adverse events [158]. Moreover, preliminary but encouraging results in the prophylaxis of migraine have been observed in a recent RCT, where the assumption of soft gelatin capsules containing nano-micellar curcumin (80 mg/day) and CoQ_10_ (300 mg/day) determined a significant reduction of frequency, severity, and duration of migraine attacks (all *p* < 0.001) [159].

#### 3.3.3. Pre-Eclampsia

Pre-eclampsia is a severe vascular complication of pregnancy. A growing collection of literature suggests that attention needs to be focused on the possible effect of CoQ_10_ during pregnancy-related hypertensive disorders [160].

Pre-eclampsia consists of the gradual development of hypertension, with values of SBP >140 mmHg and/or diastolic blood pressure (DBP) > 90 mmHg. However, in some cases, there is worsening of preexisting hypertension, generalized edema, proteinuria (300 mg/L or more in 24 h), and sometimes blood clotting disorders that arise after 20 weeks of gestation [161]. Oxidative stress could be one of the causing factors of this dangerous condition [162]. From one side, pregnant women with established pre-eclampsia have significantly lower plasma levels of CoQ_10_ compared to healthy pregnant women [163,164]. A single trial in which CoQ_10_ has been assumed at the dose of 200 mg/day for 20 weeks until delivery concluded with a reduction of the risk of developing pre-eclampsia in women at risk for the condition (*p* = 0.035) [165]. However, a recent meta-analysis of twenty-nine RCTs highlighted that the antioxidant strategy, both by using of CoQ_10_ and by using of other agents (vitamins, resveratrol, or/and arginine), did not exert significant beneficial effects on maternal and fetal outcomes [166]. Further research is needed in this field.

#### 3.3.4. CoQ_10_ and Statin-Intolerance

Statin-associated myopathy pathogenetic mechanisms are still not fully understood. The most probable hypotheses are related to the increased intracellular lipid production and lipid myopathy, decreased sarcolemmal cholesterol, and reduction in small guanosine triphosphate-binding proteins and in mitochondrial CoQ_10_ [167]. Statins, the milestone in lipid-lowering treatment, inhibit hydroxyl-methylglutaryl coenzyme A (HMG-CoA) reductase, a rate limiting enzyme not only in cholesterol synthesis but also in the synthesis of farnesyl pyrophosphate that is essential for CoQ_10_ biosynthesis, thus explaining the link between statin use and CoQ_10_ deficiency [168]. In fact, a recent meta-analysis of 12 RCTs involving 1776 participants concluded that, compared to the placebo, statin treatment resulted in a reduction of circulating CoQ_10_ (SMD −2.12; 95% CI −3.40 to −0.84; *p* = 0.001) independently from statin solution, intensity, and treatment time [169].

No study has yet been designed to demonstrate that CoQ_10_ supplementation could prevent statin-related myalgia. However, a meta-analysis of 12 RCTs involving 575 patients concluded that, compared to the placebo, CoQ_10_ supplementation ameliorated statin-associated muscle symptoms, such as muscle pain (weighted mean difference (WMD) −1.60; 95% CI −1.75 to −1.44; *p* < 0.001), muscle weakness (WMD −2.28; 95%CI −2.79 to −1.77; *p* = 0.006), muscle cramping (WMD −1.78; 95% CI −2.31 to −1.24; *p* < 0.001), and muscle tiredness (WMD −1.75; 95% CI −2.31 to −1.19; *p* < 0.001), whereas no reduction in plasma creatine kinase levels was observed after CoQ_10_ supplementation (WMD 0.09; 95% CI −0.06 to 0.24; *p* = 0.23) [170]. These positive effects are usually achieved only with high dosages of CoQ_10_ (≥200 mg/day).

However, CoQ_10_ could have a positive impact on the management of patients more likely to develop statin-related side effects. In fact, it has been clinically proven that CoQ_10_ supplementation could be able to improve self-perceived fatigue in healthy subjects, [171] in obese patients [172], and in patients affected by fibromyalgia [173,174] even if larger RCTs are needed to confirm this preliminary data.

## 4. Discussion

Theoretically, CoQ_10_ is an ideal dietary supplement. It is contained in some foods, its dosage in blood is feasible, and its deficiency is associated with some diseases, while its supplementation tends to restore a physiological condition (Table 2). Moreover, the supplementation with CoQ_10_ is safe, even with chronic exposure to 900 mg/day [175] and in frail patients, like elderly and CKD patients, without any known pharmacological interactions [3].

**Figure 3:**
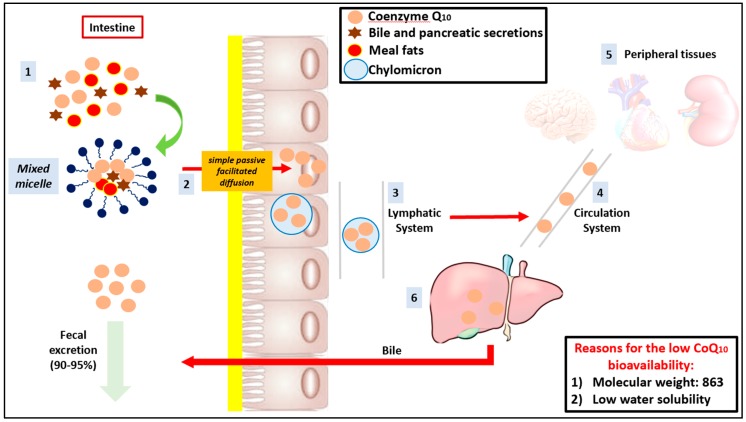
Coenzyme Q_10_ physiology: (1) CoQ_10_ arrives in intestinal lumen with exogenus cholesterol after a meal (if administered in fed state. (2) CoQ_10_ is taken up from the mixed micelles, together with the meal fats and bile and pancreatic secretions, which facilitate its solubilization and the entrance in the enterocytes via the simple passive facilitated diffusion. (3) CoQ_10_ is incorporated in the chylomicrons and subsequently reaches the bloodstream through the lymphatic system. (4) Through the bloodstream, CoQ_10_ is distributed to peripheral tissues (5) and to the liver (6), where it is partially re-excreted in the bile and eliminated with the faeces.

The results derived from clinical trials testing the efficacy of CoQ_10_ supplementation in different settings are often contrasting and complicate the process of making definitive conclusion on its efficacy in a number of conditions. This is due to a series of causes: the studies are often underpowered, the duration is too short to test the effects on hard outcomes, the methodology applied is sometime of low quality with a scarce standardization of patients characteristics at the baseline, the tested dosage is not titrated based on the blood CoQ_10_ level, and there is usually no quantification of CoQ_10_ intake with diet (even if this is usually very low). However, one of the most important problems about CoQ_10_ is related to its poor oral bioavailability. In fact, most of the CoQ_10_ integrated is eliminated through the faeces and only a fraction of that supplement reaches the blood and thus the tissues and organs [176]. CoQ_10_ is a molecule with relatively high molecular weight (MW = 863) and is insoluble in water. Because of these reasons, it is poorly absorbed in the gastrointestinal tract, and the key to effective supplementation is therefore the improvement of its bioavailability [177]. Intestinal absorption of CoQ_10_ occurs firstly through the emulsification and formation of “mixed micelles” with fatty meal constituents, also facilitated by bile and pancreatic secretions in the small intestine. It is therefore important to stress that the assumption of CoQ_10_ in fed state can significantly improve its absorption [178]. The absorption efficiency is well known to be dose dependent and occurs through a “simple passive facilitated diffusion” process: “passive” because it does not require the use of energy and “facilitated” because the intestinal transport is made possible by a lipid carrier, which is usually a monoglyceride fat [179]. In the enterocytes, CoQ_10_ is incorporated into chylomicrons and subsequently reaches the bloodstream through the lymphatic system (Figure 3). The results of pharmacokinetic studies conducted using deuterium-labeled CoQ_10_ [180] demonstrated slow absorption in the gastrointestinal tract (T_max_ ≈ 6 h) with a second plasma peak observed approximately 24 h after the oral intake [179]. This second peak could be attributed to both enterohepatic recirculation and hepatic redistribution of the circulation, mainly through the LDL/VLDL fractions [178].

To date, various formulations and dosages of CoQ_10_ are present on the market, such as tablets, chewable tablets, capsules, and gels containing oily suspensions. However, the oral bioavailability of this supplement is extremely variable in relation to many aspects. For example, the type of formulation and the release method, the dosage of CoQ_10_, and the mode of administration (e.g., with or without water and before or after meals) are biopharmaceutical factors that may affect bioavailability, as highlighted before [181]. Regarding the molecule, the ubiquinol form is the most available compared to ubiquinone, in particular if supplemented in fed state and conveyed through specific strategies like the use of liposomes, nano-emulsions nanostructured lipid carriers, and micelles [182,183]. The reduction of particle size (including the use of nanoparticles), the use of oily suspensions, and the solubilization and increase of solubility in water are also viable strategies [184]. In particular, the CoQ_10_ and β-cyclodextrin complex has been developed in addition to the intention of improving solubility in water to implement the technological properties and stability of CoQ_10_ [185], permitting the preparation of aqueous formulations, such as syrups. The improvement of bioavailability with CoQ_10_ + β-cyclodextrins and with ubiquinol have already been demonstrated in humans [186,187,188], with satisfactory results. Table 3 summarizes the main biopharmaceutical strategies used to increase the bioavailability of CoQ_10_.

Even though these formulations allow an important increase of bioavailability, it is important to underline that most of the orally supplemented CoQ_10_ is eliminated via faeces [175]. Furthermore, CoQ_10_ exerts many mild positive effects on different tissues and metabolism. They could individually not be so relevant from a quantitative point of view, but it is really difficult to quantify their impact as a whole on human health. In fact, the long-term contemporary reduction of systemic inflammation and oxidative stress, a mild reduction of blood pressure, and insulin-resistance could have positive impacts on cardiovascular disease risk.

## 5. Conclusions

Clinical evidence supports supplementation with high doses of bioavailable-CoQ_10_ (≥200 mg/day) to support heart health in patients affected by coronary heart disease and heart failure, partly modulating a number of risk factors for these conditions, and partly directly acting on myocardial cell metabolism. Long-term RCTs are still needed to confirm and better understand the efficacy and safety profile of this molecule in a large number of patients and CV diseases.

## Figures and Tables

**Table 1:** Distribution of ubiquinone and ubiquinol in human tissues (modified from References [4,5]).

Organ	Ubiquinone Concentration (µg/g)	Ubiquinol Concentration (µg/g)
Heart	132.0	61.0
Kidneys	77.0	75.0
Liver	63.6	95.0
Muscle	39.7	65.0
Brain	13.4	23.0
Pancreas	32.7	
Spleen	24.6	
Lung	7.9	25.0
Thyroid	24.7	
Testis	10.5	
Intestine	11.5	95.0
Colon	10.7	
Ventricle	11.8	
Plasma (µmol/mL)	1.1	96.0

**Table 2:** Coenzyme Q_10_: clinical applications in cardiovascular diseases.

	Level of Evidence	Active Daily Doses	Effects on Symptoms and/or Grade of Disease	Effects on Lab or Instrumental Parameters	Effects on Hard Outcomes
**Heart Failure (HF)**	Meta-analysis of RCTs	100–300 mg	↑ self-perceived quality of life and improvement in NYHA class	↑ EF (if >30%), ↑ LVEF, ↑ CO and CI, ↑ SV, ↑ EDV, ↑ exercise capacity, ↓ ventricular arrhythmias after surgery and need of inotropic drugs (after cardiac surgery), and ↓ low-grade inflammation (TNF-alpha, IL-6, and hsCRP)	↓ MACE, total mortality, and incidence of hospital stays for HF
**Acute Myocardial Infarction (AMI)**	RCTs	120 mg	Not investigated	Prevention of alteration of the wall thickening abnormality at the infarct site and sphericity index and ↓ wall thickness opposite the site of infarction	Not investigated
**Ischemic Stroke (IS)**	RCTs	300 mg	↑ NIHSS and MMSE	Reduction of oxidative stress (?)	Not investigated
**Atrial Fibrillation (AF)**	Meta-analysis of RCTs	100–300 mg	Improvement in NYHA class, reduction of risk to develop ventricular arrhythmias, and use of inotropic drugs after surgery	Reduction of malondialdehyde and oxidative stress	Not investigated
**Cardiomyopathy**	RCTs	200–300 mg	Improvement of fatigue and dyspnea	Improvement of mean interventricular septal thickness, mean posterior wall thickness, diastolic function, and mean score for the index of cardiac failure	Not investigated
**Cardiotoxicity**	RCTs	200–300 mg	Improvement of heart’s functions (in association with L-carnitine)	Reduction of oxidative stress (nitric oxide and malondialdehyde) and ↓IL-1, TNF-α Troponin-l and Troponin-T levels (in association with L-carnitine)	Not investigated
**Hypertension**	Meta-analysis of RCTs	100–300 mg	Not reported	↑ Exercise capacity and arterial stiffness, ↑ NO bioavailability, and ↓ SBP and DBP (only in prehypertensive or hypertensive patients)	Not investigated
**Diabetes type II, Metabolic syndrome (MetS)**	RCTs	100–300 mg	Not reported	↓ Lipid peroxidation, FPG, triglycerides, and low-grade inflammation (TNF-alpha, IL-6, and hsCRP) and ↑ insulin sensitivity	Not investigated
**Dyslipidemia**	RCTs	100–300 mg	↑ self-perceived quality of life (reduction side effects of lipid-lowering drugs)	↑ Exercise capacity and arterial stiffness; ↓ lipid peroxidation, TC*, LDL-C*, TG*, BP*, FPG*, and low-grade inflammation (TNF-alpha, IL-6, and hsCRP); and ↑ insulin sensitivity *If >12 weeks of treatment	Not investigated
**Non-Alcoholic Fatty Liver Disease (NAFLD)**	Meta-analysis of RCTs	100–300 mg	Improvement in NAFLD grade	↑ Adiponectin (?) and leptin levels; ↓ AST, GGT, hsCRP, and TNF-alpha levels; and ↓ WC and lipid peroxidation	Not investigated
**Chronic Kidney Disease (CKD)**	Meta-analysis of RCTs	100–300 mg	Not investigated	↓ Lipid peroxidation, TC (?), LDL-C (?), Lp(a) (?), triglycerides (?), fasting plasma glucose (?), HbA1c (?), inflammation, and oxidative stress biomarkers (hsCRP (?) and malondialdehyde) and ↑ insulin sensitivity	Not investigated

AST = Aspartate Aminotransferase, BP = Blood Pressure, CI = Cardiac Input, CO = Cardiac Output, DBP = Diastolic Blood Pressure, EDV = End-Diastolic Volume, EF = Ejection Fraction, FPG = Fasting Plasma Glucose, GGT = Gamma-Glutamyl Transpeptidase, HF = Heart Failure, hsCRP = high sensible C-Reactive Protein, IL-6 = Interleukin 6, LDL-C = LDL-Cholesterol, Lp(a) = Lipoprotein a, LVEF = Left Ventricular Ejection Fraction, MACE = Major Adverse Cardiac Events, MMSE = Mini Mental State Examination, NIHSS = National Institute of Health Stroke Scale, NYHA = New York Heart Association, NO = Nitric Oxide, RCTs = Randomized Clinical Trials, SBP = Systolic Blood Pressure, SV = Stroke Volume, TC = Total Cholesterol, TG = triglycerides, TNF-alpha = Tumor Necrosis Factor-alpha, WC = Waist Circumference. ↓: Worsening; ↑: Improvement; ?: Unclear.

**Table 3:** Comparative study of ΔC_max_ after a single dose of different formulations of CoQ_10_ (adapted from López-Lluch et al. [188]).

Type of Formulation	Subjects	Tested Dosage	ΔCmax	Reference
Myoquinon (softgel)	Both gender (10 M, 4 F), age 18–30	100 mg	1.069	[189]
KOJ, CoQ_10_ (softgel)	Both gender (10 M, 4 F), age 18–30	100 mg	0.238	
ICT, CoQ_10_ (softgel)	Both gender (10 M, 4 F), age 18–30	100 mg	0.351	
ERG, CoQ_10_ (softgel)	Both gender (10 M, 4 F), age 18–30	100 mg	0.258	
Ubquinol QH (softgel)	Both gender (10 M, 4 F), age 18–30	100 mg	0.473	
NYD CoQ_10_ (hard gel)	Both gender (10 M, 4 F), age 18–30	100 mg	0.381	
SMF CoQ_10_	Both gender (10 M, 4 F), age 18–30	100 mg	0.181	
Capsule CoQ_10_	9 M, age 18–30	30 mg	0.31	[190]
Gelatin capsule CoQ_10_ + vitamin E	Both gender (12 M, 12 F)	100 mg	0.025	[191]
NanoSolve (purified phospholipids) capsule CoQ_10_ + vitamin E	Both gender (12 M, 12 F)	100 mg	0.103	
Capsule CoQ_10_ (powder-filled hard-shell gelatine capsule)	Both gender (3 M, 3 F), age 18–40	250 mg	0.490	[192]
Liquid (O/W liquid emulsion (20 mg/mL))	Both gender (3 M, 3 F), age 18–40	250 mg	0.980	
Chewable wafer	Both gender (15 M, 10 F), elderly people	600 mg	0.770	[193]
Chewable wafer + 300 IU vitamin E	Both gender (15 M, 10 F), elderly people	600 mg	0.660	
Softgel capsules (Mega Q-Gel “100”) CoQ_10_ solubilized in an oil-based vehicle + 900 IU d-alpha tocopherol	Both gender (15 M, 10 F), elderly people	600 mg	0.690	
Hard gelatin capsule	Both gender (15 M, 10 F), elderly people	600 mg	0.660	
Powder		333 mg	0.980	[194]
Kaneka QH, ubiquinol (softgel capsules)	Both gender (5 M, 5 F)	150 mg	1.061	[195]
	5 M	300 mg	2.506	
Chewable tablets	10 M, age 21–28	150 mg	0.120	[196]
Capsule liquid	10 M, age 21–28	150 mg	0.149	
Capsule liquid	10 M, age 21–28	150 mg	0.152	
Capsule powder	10 M, age 21–28	150 mg	0.175	
Capsule liquid	10 M, age 21–28	150 mg	0.197	
Softgel	10 M, age 21–28	150 mg	0.277	
Q-gel (CoQ_10_ solubilized in an oil-based vehicle + vitamin E) softgel	10 M, age 21–28	150 mg	0.506	
Q-gel (CoQ_10_ solubilized in an oil-based vehicle + vitamin E) softgel	8 M, age 20–26	60 mg 150 mg 300 mg	0.267 0.802 1.010	
Softgel CoQ_10_	36 M, age 18–40	100 mg	0.259	[197]
Softgel CoQ_10_ + sterols	36 M, age 18–40	100 mg	0.189	
Hardgel (CoQ_10_ + 400 mg 400 mg of Emcompress)	Both gender (5 M, 5 F), age 24–30	100 mg	0.775	[180]
Softgel Bioqinon (CoQ_10_ + 400 mg of soybean oil)	Both gender (5 M, 5 F), age 24–30	100 mg	1.454	
Softgel (CoQ_10_ + 20 mg of polysorbate 80, 100 mg of lecithin + 280 mg of soybean oil)	Both gender (5 M, 5 F), age 24–30	100 mg	0.837	
Softgel (CoQ_10_ + 20 mg of polysorbate 80 + 380 mg of soybean oil)	Both gender (5 M, 5 F), age 24–30	100 mg	0.883	

Myoqinon (soy-oil matrix, drug specification heat/cooling recrystallization procedure); KOJ, CoQ_10_ (same as Myoqinon but without heat/cooling procedure); ICT, CoQ_10_ (olive oil, cocoa-butter produced accordingly normal softgel filling technology); ERG, CoQ_10_ (olive oil, cocoa-butter, 25 mg vitamin C produced accordingly normal softgel filling technology); Ubiqinol QH (MCT-oil, 12 mg vitamin C); NYD, CoQ_10_ (fine grinded (micronized) CoQ_10_ powder); SMF, CoQ_10_ (olive-oil/soy-oil matrix produced accordingly normal softgel filling technology); NanoSolve (Lipoid GmbH, Ludwigshafen, Germany); Kaneka QH (ubiquinol emulsified with diglycerol monooleate, rapeseed oil, soy lecithin, and beeswax).
